# Rethinking the Top-Down Approach to Schistosomiasis Control and Elimination in Sub-Saharan Africa

**DOI:** 10.3389/fpubh.2021.622809

**Published:** 2021-02-18

**Authors:** Adeola Onasanya, Michel Bengtson, Oladimeji Oladepo, Jo Van Engelen, Jan Carel Diehl

**Affiliations:** ^1^Department of Sustainable Design Engineering, Faculty of Industrial Design Engineering, Delft University of Technology, Delft, Netherlands; ^2^Department of Parasitology, Leiden University Medical Center, Leiden, Netherlands; ^3^Department of Health Promotion and Education, Faculty of Public Health, College of Medicine, University of Ibadan, Ibadan, Nigeria

**Keywords:** schistosomiasis, control, elimination, strategies, sub-Saharan Africa

## Abstract

The control and elimination of schistosomiasis have over the last two decades involved several strategies, with the current strategy by the World Health Organization (WHO) focusing mainly on treatment with praziquantel during mass drug administration (MDA). However, the disease context is complex with an interplay of social, economic, political, and cultural factors that may affect achieving the goals of the Neglected Tropical Disease (NTD) 2021-2030 Roadmap. There is a need to revisit the current top-down and reactive approach to schistosomiasis control among sub-Saharan African countries and advocate for a dynamic and diversified approach. This paper highlights the challenges of praziquantel-focused policy for schistosomiasis control and new ways to move from schistosomiasis control to elimination in sub-Saharan Africa. We will also discuss an alternative and diversified approach that consists of a Systems Thinking Framework that embraces intersectoral collaboration fully and includes co-creating locally relevant strategies with affected communities. We propose that achieving the goals for control and elimination of schistosomiasis requires a bottom-up and pro-active approach involving multiple stakeholders. Such a pro-active integrated approach will pave the way for achieving the goals of the NTD 2021-2030 roadmap for schistosomiasis, and ultimately improve the wellbeing of those living in endemic areas.

## Introduction

Schistosomiasis is a disease of poverty affecting over 250 million people worldwide (1). It is one of the most common waterborne parasitic diseases in the world (2). There are six known species that cause schistosomiasis in humans: *Schistosoma (S) haematobium, S. mansoni, S. japonicum, S. mekongi, S. intercalatum*, and *S. guineensis*. Of these six species, *S. haematobium* and *S. mansoni* are the most commonly found species in endemic-areas, with *S. haematobium* causing urogenital schistosomiasis and *S. mansoni* causing intestinal schistosomiasis (3). *S. haematobium* has also been implicated in susceptibility to human immunodeficiency virus (HIV) (4), human papillomavirus (HPV) infections (5) and infertility (6).

Schistosomiasis is found mostly in low-income countries with the largest burden of disease in sub-Saharan Africa (1). Sub-Saharan Africa accounts for ~93% of the world's ~207 million schistosomiasis cases, with the highest prevalence found in Nigeria, Tanzania, Ghana, Mozambique, and the Democratic Republic of Congo. These 5 countries account for ~78 million cases (7, 8). Schistosomiasis commonly affects the poor living in rural, underprivileged urban or peri-urban settings with limited access to clean water, inadequate sanitation and hygiene services (9). It is also more common in fishing and agriculture dominant communities where direct interactions with water increase the risk of contracting the disease. Water-related domestic activities such as washing clothes and fetching water, as well as recreational water activities also increase the risk of infection for women and children (10).

Schistosomiasis does not only affect the health of infected persons by causing systematic and organ-specific inflammation, it also has social and economic implications for communities (7, 11). The disease is known to cause anemia, growth stunting and reduced productivity; and accounts for between 1.6 and 4.2 million disability-adjusted life years (DALYs) lost annually in sub-Saharan Africa (8, 12). Currently, the mainstay of treatment is with the use of praziquantel which is effective for the treatment of all species of schistosomiasis (7).

The World Health Organization (WHO) has developed several roadmaps for Neglected Tropical Diseases (NTDs), and many sub-Saharan African countries have made significant progress by rolling out national action plans and programmes targeting schistosomiasis control and elimination (13). Despite these efforts, schistosomiasis remains a huge problem in sub-Saharan Africa with an unmet need for treatment (14).

## Challenges With the Current Strategy for Control and Elimination of Schistosomiasis

Attempts made toward schistosomiasis control and elimination have involved several strategies ranging from disease treatment to managing complications and controlling disease transmission (2, 13, 15, 16). Schistosomiasis is currently tackled with a combination of preventive chemotherapy dispersed through mass drug administrations (MDAs), and water, sanitation, and hygiene (WASH) programs (13, 15). However, it appears that the core focus of the WHO plan for schistosomiasis control and elimination is on preventive chemotherapy, particularly MDAs in sub-Saharan Africa. Based on this stance, progress has been made on large scale treatments and partnerships with donor foundations, other international organizations and Merck, the producer of praziquantel (10, 13). Praziquantel is the drug of choice for the treatment of schistosomiasis as it has been considered cost-effective, relatively safe, inexpensive, and effective; with donor organizations willing to provide the drug at no cost (15). Despite these attributes, schistosomiasis is still highly endemic in several countries (13, 14).

This strategy of using praziquantel as the key bullet for schistosomiasis control and elimination in practice is reactive instead of proactive and is an unavoidable consequence of a one-size-fits-all approach. This reactive approach is limiting for several reasons.

First, despite efforts at making praziquantel available to those at-need and Merck KGaA's commitment to praziquantel donations, targets for MDA coverage have still not reached all people at risk who require treatment (14). This may indicate an under-representation or undercounting of cases based on low-level awareness (11, 17, 18), migratory patterns in which the disease is introduced to new or previously eliminated areas (19, 20), and an assumption of homogeneity of the disease transmission context across different regions and countries. For example, some countries such as Nigeria have prioritized praziquantel for school-aged children leaving adults and pre-school children uncovered during MDA (18). Therefore, in this context, it implies that schistosomiasis cannot be effectively eliminated in communities where MDA treatment is on-going.

Second, although there is a commitment to the donation of praziquantel, there is a high chance of recrudescence of disease to pre-MDA levels once donations reduce or cease, or even during MDA programmes (21, 22). Third, praziquantel itself has not demonstrated 100% curative ability in both single-dose and multidose regimens in various settings (23–25) implying that relying only on praziquantel treatment use during MDA is not an effective strategy for control and elimination of this disease. Fourth, given the neglected nature of the disease in most healthcare systems in sub-Saharan Africa, there is currently inadequate funding for the disease from the national governments which is likely to persist or worsen in the future once the current external funding and support reduce. There is also a potential for donor fatigue as current gains in treatment can be reversed when donation stops, because countries do not have sustainable strategies to own and incorporate programmes within their current healthcare systems (26). Lastly, the disease context is complex with an interplay of social, economic, political, and cultural factors (20, 27) that may affect achieving the goals of the NTD 2021-2030 Roadmap (28). In light of these challenges, there is a need to revisit the current top-down approach to schistosomiasis control among sub-Saharan African countries irrespective of the level of endemicity.

There have been several resolutions over time by the WHO geared toward the control and elimination of schistosomiasis including renewing interest, addressing partnerships, and in 2012, the need to attach importance to both preventative and control strategies by developing applicable plans with progressive targets (2). In 2013, the “WHA66.12 resolution” on NTDs focused on advocating for continuous country ownership of programmes for NTD prevention, control, elimination, and eradication (2, 13). The current roadmap for 2021–2030 for NTDs also reiterates the importance of community-based and applied research for effective NTD programmes. It highlights the need to integrate mainstream approaches into national healthcare systems, coordinate action across sectors (which has been challenging to operationalize), and close coordination and multisectoral action across all sectors (beyond health) (16). However, it is unclear how sub-Saharan African countries can achieve their targets beyond the desire for easy wins through the use of praziquantel as a reactive way to achieve their aims. Clearly, attaining schistosomiasis control requires a dynamic approach that incorporates more proactive and holistic strategies beyond the current top-down approach to one that incorporates the socio-cultural, epidemiological, economic and geographical dynamics within each country to create a mix-set of feasible strategies for schistosomiasis control. The uptake and domestication of these strategies will require an in-depth look into the dynamics of each region and country.

## Discussion and Recommendations

Achieving sustainable schistosomiasis control and elimination requires an innovative design that incorporates a wide range of factors and information influencing disease transmission and intervention successes, which are interdependent and interrelated, and which will benefit from a whole system context (29, 30). Therefore, we propose a proactive and dynamic approach with three broad strategies.

First, is the need to use a Systems Thinking Framework with a particular focus on medical products and technology, information and research, healthcare financing, and service delivery. This is hinged on the premise that the control and elimination of schistosomiasis, like all other NTDs, is affected by a multitude of social, cultural, economic, geographical and ecologic factors (28) which are interdependent, and for which the current use of praziquantel alone cannot solve. These interdependencies are best understood and addressed by looking at the system as a whole with a particular lens on weak points within the system (21, 26, 29).

Although the NTD 2021-2030 roadmap stresses the need for well-structured operational and implementation investigations, including community-based and applied research as the main fulcrum (16), it is still unclear how sub-Saharan countries can achieve this goal. As such, sub-Saharan countries need to identify key areas, wherein available resources can sustainably reduce schistosomiasis burden and also indirectly contribute to an improved healthcare system in the long-term. Improving access to medical products and technology includes drug procurement and supply chain for praziquantel by making it readily available for easy procurement and treatment of schistosomiasis in partnership with donors and the private sector, as well as investing in affordable, easy to use diagnostic tools which can reduce delays in accessing treatment. A number of these diagnostic tools, such as mobile phone-based technologies and rapid diagnostic tests, are either currently available or undergoing development (31–35).

There is also a need to manage information and promote research into drivers of regional and local hotspots of schistosomiasis (21, 22, 28). Service delivery has been one of the problems of schistosomiasis control in several sub-Saharan African countries with praziquantel mainly available during MDAs and the difficulty of identifying non-acute cases of urinary schistosomiasis (18). As such, we propose seeing schistosomiasis in the same light as malaria and adding regular screening at the primary care level for regions with a high prevalence to help capture those who are not covered by the MDA programmes. It is also important to capture NTDs diagnostics and treatment into current healthcare financing plans. Communities with a high incidence may benefit from specialized health insurance plans that can absorb the cost of treatment. Alternatively, it can be made mandatory through policies for coverage of NTDs by health management organizations to reduce out of pocket costs by persons with the disease. All these will require viable research with generated data used in designing effective communication interventions.

Second, strategies for schistosomiasis control and elimination should be multisectoral as the disease is not only a healthcare system problem but affects other areas of people's lives as well, such as livelihoods, recreation, and cultural practices. The physical environment is one of the key determinants of schistosomiasis infection and addressing issues related to this requires an in-depth look into sectors that relate directly to the physical environment, including socio-economic and cultural aspects (36). In this context, beyond the health ministry departments such as vector control, epidemiology, health education, medicine, nursing and pharmacology departments; other sectors/ministries/departments such as planning, statistics, community development, water resources, animal health, education, agriculture, environmental management, and finance are critical and should work together as a team. Important elements to consider for involvement include how these sectors are affected or contribute to schistosomiasis, and how strategies can be drawn up synergistically to minimize infection and re-infection and help with control. Moreover, the multisectoral team equally needs to fashion out innovative activities. For example, the promotion of fish farming and raising of shrimps that are known to eat the cercariae of schistosomes in highly endemic areas can help reduce infection rates (37) and contribute to the local economy. The introduction of shrimps that feed on the *Schistosoma* cercariae may be more useful in riverine/swampy communities, and molluscicides in inland communities and localities that do not depend on rivers for economic activities. Introducing and promoting the planting and use of natural molluscicidal agents such as soapberry Endod (*Phytolacca dodecandra*), which is also toxic to miracidia and cercariae and doubles as a natural detergent for washing clothes (38, 39) is illustrative.

Furthermore, since schistosomiasis is more common along communities situated around dams (40), a percentage of profits made from dam-derived services should be allocated for the implementation of schistosomiasis control activities. Although schistosomiasis is common in more rural areas; rural-urban migration patterns, urban planning challenges and overcrowding, and problems of rampant open defecation due to poor sanitary facilities in sub-Saharan Africa have increased the risk of schistosomiasis in urban communities implying poor urban planning. The planning departments can also collaborate with communities and community-based organizations to push for clean water and improved hygiene and sanitary services. Clean water provision, sanitation and hygiene (WASH) is critical to schistosomiasis control and elimination by preventing contaminated feces and urine from reaching open water sources such as rivers.

Third, there is a need to co-create locally relevant strategies with affected communities and regions since the burden of schistosomiasis is not equally distributed across most sub-Saharan African countries and even within countries (7, 17, 41). Therefore, affected regions and communities should be seen as collaborators in dealing with schistosomiasis control and elimination. For most control programmes, the government attempts MDA as a broad strategy without looking in-depth at the peculiarities of these communities and their challenges which can be drivers of schistosomiasis infection and burden (26, 28, 29). Thus, the current one-size-fits-all intervention using a top-down approach may be a contributor to the limited success of schistosomiasis control and elimination in sub-Saharan Africa. This is due to the complex interplay of factors and heterogeneity between individuals and their settings (28, 42, 43) making it difficult to understand drivers of schistosomiasis within high-risk communities and inability to create potentially useful and scalable solutions within these contexts. Consequently, identifying contextual problems related to schistosomiasis and developing localized solutions can go a long way in achieving schistosomiasis control and elimination solutions. In this context, the control and elimination of schistosomiasis should not just be done for the people, but with the people as the NTD 2021-2030 Roadmap has clearly highlighted ownership as being critical for schistosomiasis control and elimination. Ownership should not only be seen at the government level through policies, but there is also the need for communities to own these strategies by viewing the people in these communities as collaborators in the fight against schistosomiasis and co-creating strategies with them (44). Co-creation takes into consideration the heterogeneity (45) within countries that are based on social, behavioral, and economic factors related to infection in the at-risk population. Schistosomiasis control and elimination requires a participatory approach involving both the at-risk population and the local governance structure charting a path together for the control and elimination for communities and regions (44). For example, communities can use locally available materials and techniques such as composting toilets for improved sanitation, thus reducing open defecation and consequently reducing schistosomiasis infection. Since materials can be locally sourced and are culturally acceptable, they are more likely to be easily maintained and thus contribute to local sustainability. This can also drive a sense of ownership by communities to push for the elimination of schistosomiasis within their localities.

Co-creation has also been documented to be effective in reducing NTDs (44). The development and use of locally relevant technologies and knowledge are critical to schistosomiasis control and elimination within communities and endemic regions. Put together, the strategies from all regions then become the input to develop broad and comprehensive national policies which are locally relevant for communities. Using this proactive approach will increase the likelihood of sustainable schistosomiasis control and elimination.

## Conclusion

We propose that achieving the goals for control and elimination of schistosomiasis requires a proactive approach involving a range of stakeholders and a mixed-set of pluriform strategies that consider heterogeneity at the national and regional levels, as well as local transmission factors. These strategies should focus on locally relevant and acceptable ways to increase awareness, reduce transmission and infection, and equitable ways of treating the disease.

## Data Availability Statement

The original contributions presented in the study are included in the article/supplementary material, further inquiries can be directed to the corresponding author/s.

## Author Contributions

AO determined the overall structure of the paper with inputs from MB, OO, JD, and JV. All authors reviewed the interpretation and recommendations for critical content and read and approved the final manuscript.

## Conflict of Interest

The authors declare that the research was conducted in the absence of any commercial or financial relationships that could be construed as a potential conflict of interest.
